# Integrative multi-omics analyses identify key genes and elucidate bidirectional regulatory mechanisms in thyroid dysfunction

**DOI:** 10.1371/journal.pone.0338805

**Published:** 2026-01-22

**Authors:** Yilang Hu, Hong Hu, Lijun Xu, Xuzheng Tang, Chongyu Wang, Zhengyi Lian, Chenyu Xie, Zhiwan Xie, Qingqing Wang

**Affiliations:** 1 Department of thyroid and breast surgery, Affiliated Hospital of Nantong University, Medical School of Nantong University, Nantong City, Jiangsu Province, China; 2 Department of Medicine, Xinglin College, Nantong University, Nantong City, Jiangsu Province, China; 3 Department of thyroid and breast surgery, Changshu Hospital Affiliated to Nanjing University of Chinese Medicine, Suzhou City, Jiangsu Province, China; 4 Medical School of Nantong University, Nantong City, Jiangsu Province, China; Sreenidhi Institute of Science and Technology, INDIA

## Abstract

**Objective:**

Hyperthyroidism and hypothyroidism are globally prevalent endocrine disorders, with their pathogenesis involving multifactorial mechanisms including genetics, immunity, and metabolism. Although genome-wide association studies (GWAS) have identified risk genes such as PDE8B, critical gaps remain in the annotation of causal variants in non-coding regions, characterization of tissue-specific regulatory networks, and understanding of ethnic heterogeneity. This study aimed to systematically identify genes associated with hyperthyroidism and hypothyroidism and unravel their underlying molecular mechanisms through multi-omics integration.

**Methods:**

We included data from the ThyroidOmics Consortium, comprising 1,840 hyperthyroidism cases (49,983 controls) and 3,340 hypothyroidism cases (49,983 controls). Core candidate genes were prioritized using a combination of SMR-HEIDI analysis, cross-tissue transcriptome-wide association study (TWAS), mBAT-combo rare variant analysis, and polygenic priority score (PoPS). GTEx colocalization (coloc) analysis was used to validate tissue-specific colocalization between these candidate genes and disease signals. Phenome-wide association study (PheWAS), KEGG pathway enrichment, and protein-protein interaction (PPI) network analyses were performed to explore gene functions, with potential targeted drugs predicted using the Drug Signatures Database (DSigDB).

**Results:**

Cross-validation by four methods identified FAM227B, PDE8B, and PDE10A as key genes for hyperthyroidism, and PDE8B as the critical gene for hypothyroidism. GTEx coloc analysis (with PP4 > 0.8 as the threshold) confirmed significant colocalization: FAM227B with hyperthyroidism signals in the adrenal gland, lung, and minor salivary gland; PDE8B with both hyperthyroidism and hypothyroidism signals in thyroid tissue; and PDE10A with hyperthyroidism signals in thyroid tissue. As a core member of the phosphodiesterase family, PDE8B exhibited bidirectional regulatory characteristics in thyroid hormone synthesis via the cAMP signaling pathway and nucleotide metabolism network: its inhibition promoted hormone synthesis in hypothyroidism, while its interaction with PDE10A suppressed overactive cAMP signaling in hyperthyroidism. PheWAS linked FAM227B to cardiovascular diseases and PDE10A to neurological pathways. KEGG enrichment analysis highlighted the “morphine addiction” pathway (p = 6.12 × 10 ⁻ ⁵), suggesting potential neuroendocrine crosstalk. Notably, potential drugs targeting FAM227B, PDE8B, and PDE10A were identified.

**Conclusion:**

Through multi-omics integration, this study identifies PDE8B as a central gene associated with thyroid dysfunction, characterized by tissue-specific colocalization, and elucidates its critical roles in signaling pathways, comorbidity associations, and drug targeting. These findings provide insights into the bidirectional regulatory mechanisms of hyperthyroidism and hypothyroidism and a theoretical basis for developing phosphodiesterase family-based precision therapies. It should be noted that all samples in this study are of European ancestry, and the generalizability of the results in other ethnic groups remains to be verified.

## 1. Introduction

Hyperthyroidism and hypothyroidism are clinically common endocrine disorders with complex pathogenesis, resulting from the interplay and interaction of genetic, immune, and environmental factors [1]. Globally, their prevalence rates are 0.2%−1.3% and 0.3%−4.6%, respectively [2,3]. These two conditions not only cause imbalances in thyroid hormone metabolism but also trigger a cascade of reactions, being closely associated with multisystem complications such as cardiovascular diseases and neurological dysfunctions [4]. Data from family and twin studies strongly indicate that genetic factors play a pivotal role in the pathogenesis of hyperthyroidism (35%−60%) and hypothyroidism (50%−70%), with significant contributions to their pathogenesis [5]. This finding suggests that in-depth exploration of their genetic mechanisms is crucial for precision diagnosis and treatment.

Although genome-wide association studies (GWAS) have identified risk genes such as PDE8B and PTPN22, existing research faces multiple challenges. On one hand, most risk loci are located in non-coding regulatory regions, and due to complex linkage disequilibrium (LD) patterns, the functional annotation of causal variants and tissue-specific expression regulatory mechanisms of gene expression remain unclear [6]. On the other hand, hyperthyroidism and hypothyroidism exhibit bidirectional opposition in pathological manifestations but may share some molecular pathways (e.g., cAMP signaling), making it difficult for traditional single-gene analyses to unravel the dynamic molecular mechanisms of such bidirectional regulation [7]. Additionally, existing studies have mostly focused on European populations, and the impact of trans-ethnic genetic heterogeneity on disease susceptibility requires more extensive validation.

To address these limitations, this study integrates multi-omics data and interdisciplinary analytical approaches, establishing a comprehensive research framework encompassing “gene screening—mechanism elucidation —drug prediction.” Through SMR-HEIDI analysis to distinguish gene pleiotropy from linkage effects, combined with cross-tissue transcriptome-wide association study (TWAS), mBAT-combo rare variant detection, and polygenic priority score (PoPS)for gene prioritization, we systematically screen for key candidate genes for hyperthyroidism and hypothyroidism. Further, phenome-wide association study (PheWAS), KEGG pathway enrichment analysis, and protein-protein interaction (PPI) network analysis are used to elucidate gene functions and potential pathways, with targeted drugs predicted based on the DSigDB database. This study aims to elucidate the genetic regulatory network of thyroid dysfunction and provide new targets for the development of precision therapeutic strategies.

## 2. Materials and methods

**Fig 1:**
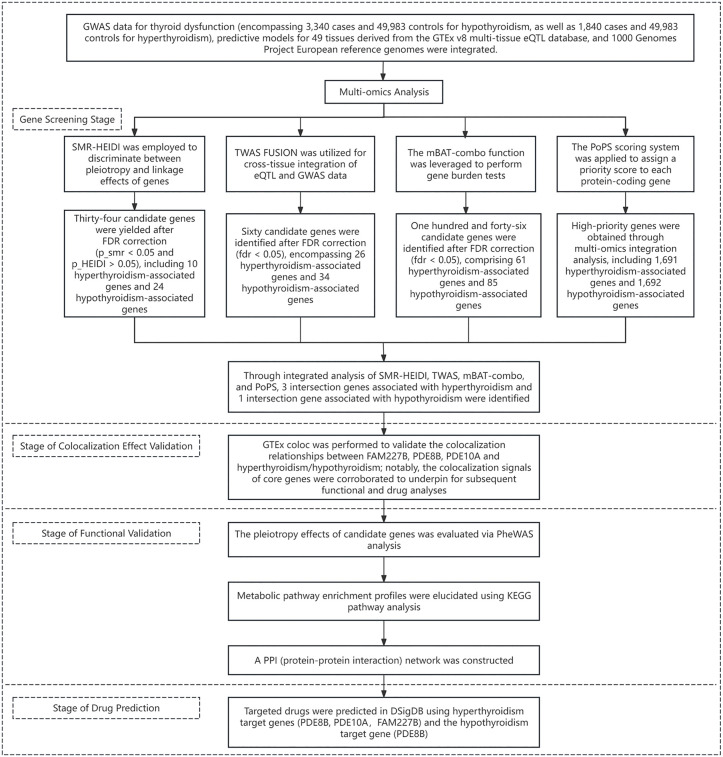
Overview of the study design. Abbreviations: GTEx, Genotype-Tissue Expression; FDR, false discovery rate; SMR, Summary-data-based Mendelian Randomization; HEIDI, Heterogeneity in Dependent Instruments; TWAS, Transcriptome-Wide Association Study; FUSION, Functional Summary-based Imputation; mBAT, Multivariate Set-Based Association Test; PoPS, Polygenic Priority Score; GTEx coloc, colocalization analysis based on GTEx data; PheWAS, Phenome-Wide Association Study; KEGG, Kyoto Encyclopedia of Genes and Genomes (pathway enrichment analysis); PPI, protein-protein interaction; DSigDB, Drug Signatures Database.

The analytical process is illustrated in Fig 1. First, expression quantitative trait locus (eQTL) data were collected from the Genotype-Tissue Expression (GTEx) v8 dataset, and thyroid dysfunction data were retrieved from the ThyroidOmics Consortium. Subsequently, candidate genes were screened using SMR-HEIDI, transcriptome-wide association study (TWAS), multivariate set-based association test (mBAT)-combo, and polygenic priority score (PoPS) methods. After identifying genes intersecting across the four methods, phenome-wide association study (PheWAS), KEGG pathway enrichment, and protein-protein interaction (PPI) network analyses were performed to comprehensively evaluate the selected genes. Finally, targeted drugs for the intersecting genes were predicted using the Drug Signatures Database (DSigDB).

### 2.1. Data sources

This study included the GWAS dataset released by the ThyroidOmics Consortium (https://genepi.med.uni-greifswald.de/thyroidomics/) in 2018, comprising 1,840 hyperthyroidism cases with 49,983 controls and 3,340 hypothyroidism cases with 49,983 controls. All samples were of European ancestry, excluding individuals with concurrent severe liver/kidney diseases or autoimmune disorders. Gene expression data were derived from the GTEx v8 dataset [8], which includes 17,382 samples across 49 tissues. The genetic variant mapping was referenced to the 1000 Genomes Project Phase 3 data from European populations to ensure the accuracy of genetic variant localization.(S1)

### 2.2. Candidate gene screening

Briefly, the SMR and HEIDI methods integrated summary-level data from GWAS and eQTL studies to test whether transcripts and phenotypes are associated due to shared causal variants (i.e., pleiotropy). Compared with similar integrative approaches, SMR offers the advantage of distinguishing between pleiotropic models (where gene expression and phenotype are related via a single shared genetic variant) and linkage models (where two or more distant genetic variants in linkage disequilibrium [LD] independently affect gene expression and phenotype) [9–11]. In this study, the SMR-HEIDI analysis was performed with the following parameters: the cis-eQTL window was set to 2000 Kb (i.e., ± 1 Mb upstream and downstream of candidate genes), the top associated eQTLs for SMR testing were filtered using the default p-value threshold (--peqtl-smr = 5.0e-8); eQTLs involved in the HEIDI test were selected based on the default threshold (--peqtl-heidi = 1.57e-3, corresponding to a chi-square value of 10 with df = 1), and genes meeting p_smr < 0.05 and p_HEIDI > 0.05 after FDR correction were defined as candidate genes.[12].

We performed TWAS using the FUSION tool (http://gusevlab.org/projects/fusion/) by integrating thyroid dysfunction GWAS data with eQTL data from 49 tissues in GTEx v8 to assess the association between each gene and the disease [13,14]. Initially, LD between the prediction model and SNPs at each GWAS locus was estimated using 1000 Genomes European samples. FUSION then integrated multiple predictive models (BLUP, BSLMM, LASSO, Elastic Net, and top 1) to evaluate the overall impact of SNPs on gene expression weights. The model with the highest predictive performance was subsequently used to determine gene weights [15]. On this basis, we combined the genetic effects of hyperthyroidism and hypothyroidism (GWAS Z-scores) with these gene weights to conduct TWAS for hyperthyroidism and hypothyroidism, with results included if they met an FDR < 0.05 in cross-tissue analysis.

In this study, we employed the mBAT-combo function of GCTA software for analyses—a sophisticated statistical method designed to detect masking effects among multiple SNPs in the context of linkage disequilibrium (LD). It integrates mBAT and fastBAT to enable comprehensive profiling of SNP sets, thereby identifying genetic signals that are frequently missed by traditional single-SNP analyses. For the definition of SNP sets in mBAT-combo, the analysis region for each gene was restricted to ±50 Kb around the gene body. During SNP filtering, variants with excessive differences in the frequency of the effect allele (A1) between GWAS summary data and the LD reference dataset were excluded based on an allele frequency difference threshold of 0.2. In fastBAT analysis, LD pruning was performed using an LD r² threshold of 0.9. For the mBAT test, the minimum proportion of total variance in the LD matrix explained by principal components was set to 0.9. Finally, the SNP sets included in the analyses were output, and results were reported with a false discovery rate (FDR) < 0.05 as the significance criterion [16–18].

The polygenic priority score (PoPS) is built on data from extensive tissue and single-cell expression datasets, curated biological pathways, and predicted protein-protein interaction data [12]. It assigns a priority score to each protein-coding gene based on the enrichment of these datasets, and we used PoPS to select the top 10% of genes rated as relevant to hyperthyroidism and hypothyroidism.

### 2.3. Colocalization analysis

To validate the colocalization characteristics between core candidate genes (FAM227B, PDE8B, PDE10A) and thyroid dysfunction (hyperthyroidism/hypothyroidism), and to distinguish between “shared genetic variants” and “non-specific associations caused by linkage disequilibrium (LD)” for excluding linkage effect interference, this study employed GTEx data-based colocalization analysis (GTEx coloc).

This analysis is based on a Bayesian statistical model, which systematically evaluates the likelihood of genetic signal colocalization by quantifying posterior probabilities (PP) [19]. The core logic is that if disease-associated signals (GWAS) and gene expression regulatory signals (eQTL) originate from the same genetic variant, their significance distributions in genomic positions should show statistical association. Two types of core data were integrated in the analysis: ① Summary statistics of thyroid dysfunction GWAS (hyperthyroidism: 1,840 cases vs 49,983 controls; hypothyroidism: 3,340 cases vs 49,983 controls); ② Tissue-specific eQTL data from the GTEx v8 dataset (covering 49 tissues and restricted to the ± 1 Mb region flanking candidate genes)

Colocalization calculations were implemented using the coloc R package [19,20], which quantifies posterior probabilities (PP) by constructing five potential models: PP0 (no association), PP1 (GWAS signal only), PP2 (eQTL signal only), PP3 (two signals driven by independent genetic variants), and PP4 (both signals driven by a single genetic variant). This study focused on PP4, as it directly reflects the probability that “disease and gene expression are regulated by the same genetic variant” and serves as the core indicator for determining colocalization. Referring to the classic threshold for colocalization studies (PP4 > 0.8, indicating high-confidence colocalization), core genes with both genetic association and functional regulatory evidence were finally screened, providing functional priority support based on colocalization effects for subsequent analysis of their specific regulatory mechanisms in thyroid dysfunction [21].

### 2..4. Phenome-Wide Association Study

To further evaluate the horizontal pleiotropy of potential drug targets and possible side effects, a phenome-wide association study (PheWAS) was conducted on the AstraZeneca PheWAS Portal (https://azphewas.com/) [22].

### 2.5. Enrichment analysis

To investigate the functional characteristics and biological relevance of the identified prospective therapeutic target genes, pathway analysis was performed using the Enrichr online platform. KEGG pathways can provide information on metabolic pathways [23].

### 2.6. Construction of protein-protein interaction network

Evaluating and analyzing the protein-protein interaction (PPI) network can enhance the understanding of intracellular protein interaction patterns. In this study, the PPI network was constructed using the STRING database, with a confidence score of 0.4 set as the minimum required interaction score (chosen based on prior studies focusing on thyroid-related protein interactions to enhance the specificity of functional associations) and all other parameters remained at default settings [24]. PPI results were further visualized using Cytoscape (V3.9.1) [25]. Additionally, GeneMANIA (https://genemania.org/)was used for PPI analysis [26].

### 2.7. Candidate drug prediction

To evaluate the therapeutic target potential of the core genes identified in this study (hyperthyroidism: FAM227B, PDE8B, PDE10A; hypothyroidism: PDE8B), we performed potential protein-drug interaction analyses using the Drug Signature Database (DSigDB, http://dsigdb.tanlab.org/DSigDBv1.0/) [27]. This database integrates association information across 22,527 gene sets, 19,531 genes, and 17,389 compounds, providing a reliable resource for systematically exploring regulatory relationships among drugs, chemicals, and target genes. In the analysis, we first uploaded the core target genes to DSigDB and screened candidate drugs based on the database’s built-in gene-drug association algorithm. We further introduced a “combined score” to quantify the reliability and clinical translation value of drug-target gene associations, with the calculation formula defined as: Combined Score = (-log₁₀(adjusted P-value) × Odds Ratio) × Gene Expression Regulatory Weight. Herein, the adjusted P-value and Odds Ratio were directly derived from DSigDB’s statistical analysis results, reflecting the statistical significance and effect strength of drug-target gene associations, respectively. The gene expression regulatory weight was set based on the standardized Z-scores of target gene expression levels in the thyroid gland and disease-related tissues (e.g., adrenal gland, pituitary gland) from the GTEx v8 dataset (range: 1–5), with higher weights assigned to target genes with higher expression levels and stronger tissue specificity in key tissues. This scoring system comprehensively considers the credibility of drug-target associations, the efficacy of drug actions, and the functional relevance of target genes in the pathological mechanisms of thyroid dysfunction, thereby providing a quantitative basis for the prioritization of candidate drugs and the evaluation of their clinical translation potential in subsequent studies.

## 3. Results

### 3.1. Candidate gene screening

**Fig 2:**
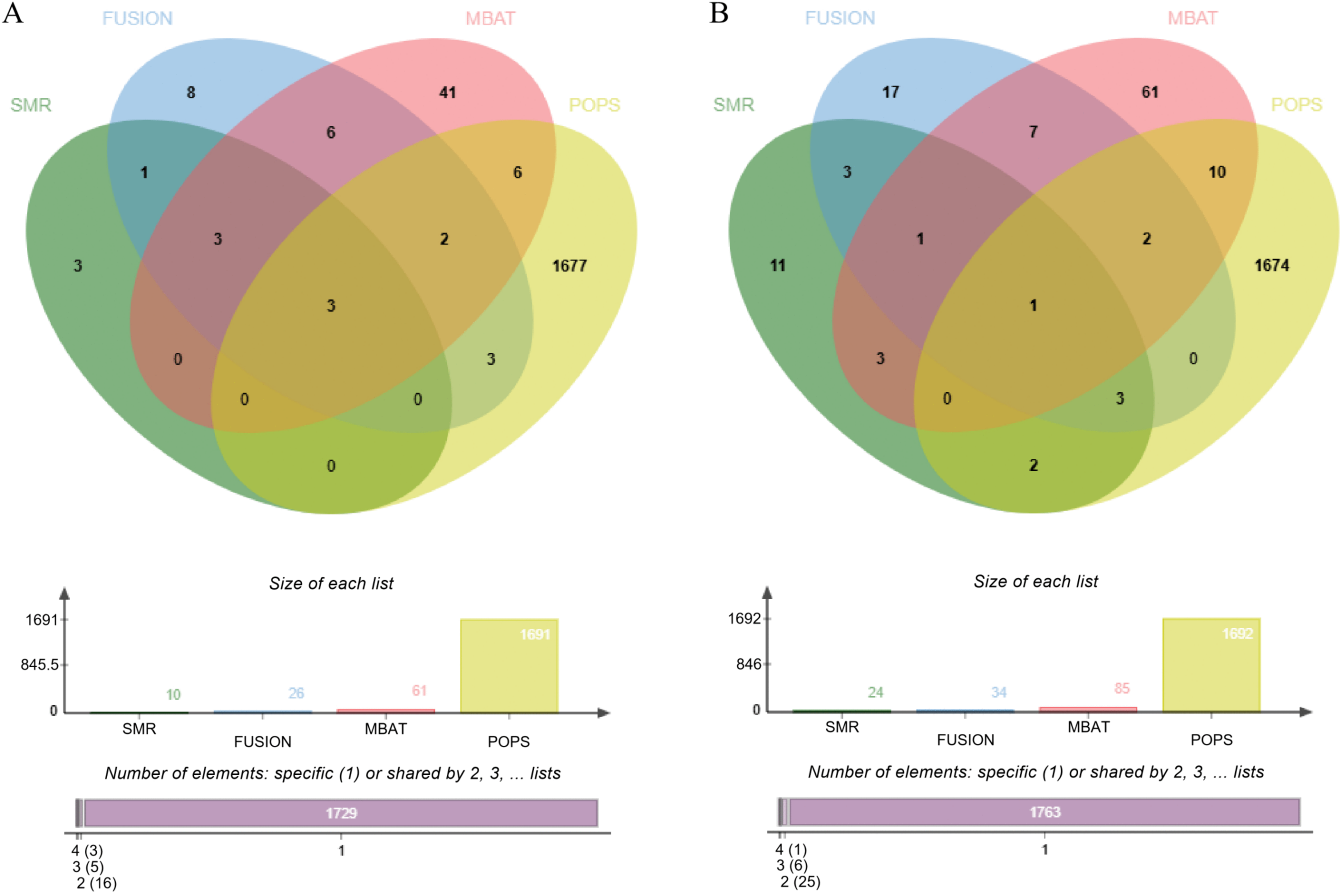
Venn diagrams illustrating candidate genes for hyperthyroidism (A) and hypothyroidism (B) identified via four approaches. Abbreviations: SMR, Summary-data-based Mendelian Randomization; FUSION, Functional Summary-based Imputation;mBAT, Multivariate set-based association test;PoPS, Polygenic Priority Score. Notably, the intersection genes were validated through multiple assessments (FDR < 0.05), which suggests a core role of PDE8B in thyroid dysfunction. Conversely, FAM227B and PDE10A were identified as hyperthyroidism-specific candidate genes.

In this study, four gene prioritization and association analysis methods—SMR, TWAS, mBAT-combo, and PoPS—were used to screen genes associated with hyperthyroidism and hypothyroidism in the ThyroidOmics Consortium dataset. Among these, SMR identified 10 hyperthyroidism-related genes and 24 hypothyroidism-related genes; TWAS identified 26 hyperthyroidism-related genes and 34 hypothyroidism-related genes; mBAT-combo identified 61 hyperthyroidism-related genes and 85 hypothyroidism-related genes; and PoPS identified 1691 hyperthyroidism-related genes and 1692 hypothyroidism-related genes. Through cross-validation of the four methods, 3 intersecting genes for hyperthyroidism (FAM227B, PDE8B, PDE10A; Fig 2A) and 1 intersecting gene for hypothyroidism (PDE8B; Fig 2B) were finally identified. Notably, PDE8B was consistently confirmed by all four methods (Table 1).

**Table 1:** Number of candidate genes screened by four methods and results of intersections.

Methods	Number of hyperthyroidism candidate genes	Number of hypothyroidism candidate genes
SMR-HEIDI	10	24
TWAS-FUSION	26	34
mBAT-combo	61	85
PoPS	1691	1692
Final intersection	3	1

### 3.2. SMR

Analysis using SMR and HEIDI methods identified a total of 10 hyperthyroidism-related genes and 24 hypothyroidism-related genes.

Among hyperthyroidism-related genes, both PDE8B (phosphodiesterase 8B) and PDE10A (phosphodiesterase 10A) showed significant negative correlations (associated with reduced hyperthyroidism risk): the SMR effect size for PDE8B was −0.638 (P_SMR = 5.78 × 10 ⁻ ¹⁶), and for PDE10A was −0.505 (P_SMR = 6.78 × 10 ⁻ ⁷). This suggests that their high expression may reduce the risk of hyperthyroidism by inhibiting excessive activation of the cAMP signaling pathway and reducing abnormal activation of thyroid cells. Additionally, FAM227B also showed a negative correlation (β_SMR = −0.890, P_SMR = 5.89 × 10 ⁻ ⁶), and its role may be indirectly related to metabolic regulation of thyroid cells or angiogenic signaling.

Among hypothyroidism-related genes, PDE8B showed a significant positive correlation (associated with increased hypothyroidism risk, β_SMR = 0.290, P_SMR = 8.33 × 10 ⁻ ⁸), in striking contrast to its negative correlation in hyperthyroidism (reducing risk). This suggests that its high expression may accelerate cAMP degradation, thereby inhibiting thyroid hormone synthesis and increasing the risk of hypothyroidism. The key enzyme gene TPO (thyroid peroxidase) showed a positive correlation (β_SMR = 0.389, P_SMR = 1.95 × 10 ⁻ ⁹), suggesting that decreased expression of this gene is associated with increased hypothyroidism risk, possibly by directly affecting iodine activation and thyroxine production. The immune-related gene HLA-DQB1 (human leukocyte antigen DQB1) showed a significant negative correlation signal (β_SMR = −0.126, P_SMR = 2.65 × 10 ⁻ ⁵). It should be clarified that this negative correlation indicates that its high expression is associated with reduced hypothyroidism risk (protective effect), while low expression is associated with increased hypothyroidism risk (risk factor), which is consistent with the biological meaning of the negative β_SMR value. It is speculated that this gene may be involved in thyroid tissue damage by regulating immune cell activation, i.e., low expression is more likely to promote the development of hypothyroidism.

### 3.3. TWAS fusion

We conducted cross – tissue Transcriptome - Wide Association Study (TWAS) on genes associated with hyperthyroidism and hypothyroidism using the FUSION tool. False Discovery Rate (FDR) correction was applied to the gene data of each tissue, and genes with FDR less than 0.05 were selected.

A total of 26 genes were included in the analysis of hyperthyroidism-associated genes. After FDR correction (FDR < 0.05), several genes exhibited distinct tissue-specific expression signatures. For instance, PDE8B showed a significant positive association in thyroid tissue (TWAS.Z = 5.70217, FDR = 5.71002e-05) and pituitary tissue (TWAS.Z = 4.213, FDR = 1.89e-04)—as the central organ for thyroid hormone synthesis and secretion, the thyroid gland directly contributes to the pathophysiology of hyperthyroidism, while the pituitary gland regulates thyroid function via secretion of thyroid-stimulating hormone (TSH), together forming a key axis for thyroid hormone homeostasis. We hypothesize that the positive association signal of PDE8B may be implicated in the remodeling of hormone feedback regulatory pathways during hyperthyroidism. This observation contrasts with previous reports where PDE8B typically showed a significant negative association with hyperthyroidism-related genes, indicating that its regulatory mechanism is likely tissue-dependent and context-specific, with heterogeneous functional modalities across different physiological and pathological states. On the other hand, FAM227B displayed negative association signals in both the adrenal gland (TWAS.Z = −4.74631, FDR = 2.07e-06) and arterial tissues (TWAS.Z = −4.54346, FDR = 5.53e-06). Its encoded protein may participate in angiogenesis regulation, indirectly modulating the microenvironmental homeostasis of thyroid follicular cells.

In the analysis of hypothyroidism – associated genes, 34 genes were involved. Following FDR correction (FDR < 0.05), several genes stood out. NBL1 in coronary artery tissue showed a significant positive association (TWAS.Z = 6.0829, FDR = 1.18e - 09), and VAV3 in the adrenal gland also presented a significant positive correlation (TWAS.Z = 4.701879, FDR = 2.58e - 06). Although thyroid peroxidase (TPO) did not display prominent significant associations in the current dataset, given its well – established role as a key enzyme in thyroid hormone synthesis and its previously reported significant positive association (e.g., TWAS.Z = 6.06, FDR = 1.35 × 10 ⁻ ⁹), it remains a strong candidate as an important genetic factor in the pathogenesis of hypothyroidism.

### 3.4. MBAT-combo

In this study, we leveraged the mBAT-combo function within the GCTA software for preliminary analyses, followed by joint validation and screening of results through the integration of mBAT and fastBAT methodologies. After false discovery rate (FDR) correction (FDR < 0.05), a total of 61 hyperthyroidism-associated candidate genes and 85 hypothyroidism-associated candidate genes were identified.

### 3.5. Polygenic priority score

Polygenic priority score (PoPS) was applied to analyze genes associated with hyperthyroidism and hypothyroidism. A large amount of gene information was initially retrieved from the dataset; after PoPS-based screening, the top 10% ranked genes were selected to define gene sets significantly linked to the two conditions. Among the filtered genes, 1691 hyperthyroidism-related and 1692 hypothyroidism-related genes with significant PoPS scores were identified, which may play important roles in the pathogenesis of hyperthyroidism and hypothyroidism.

### 3.6. Colocalization analysis

**Fig 3:**
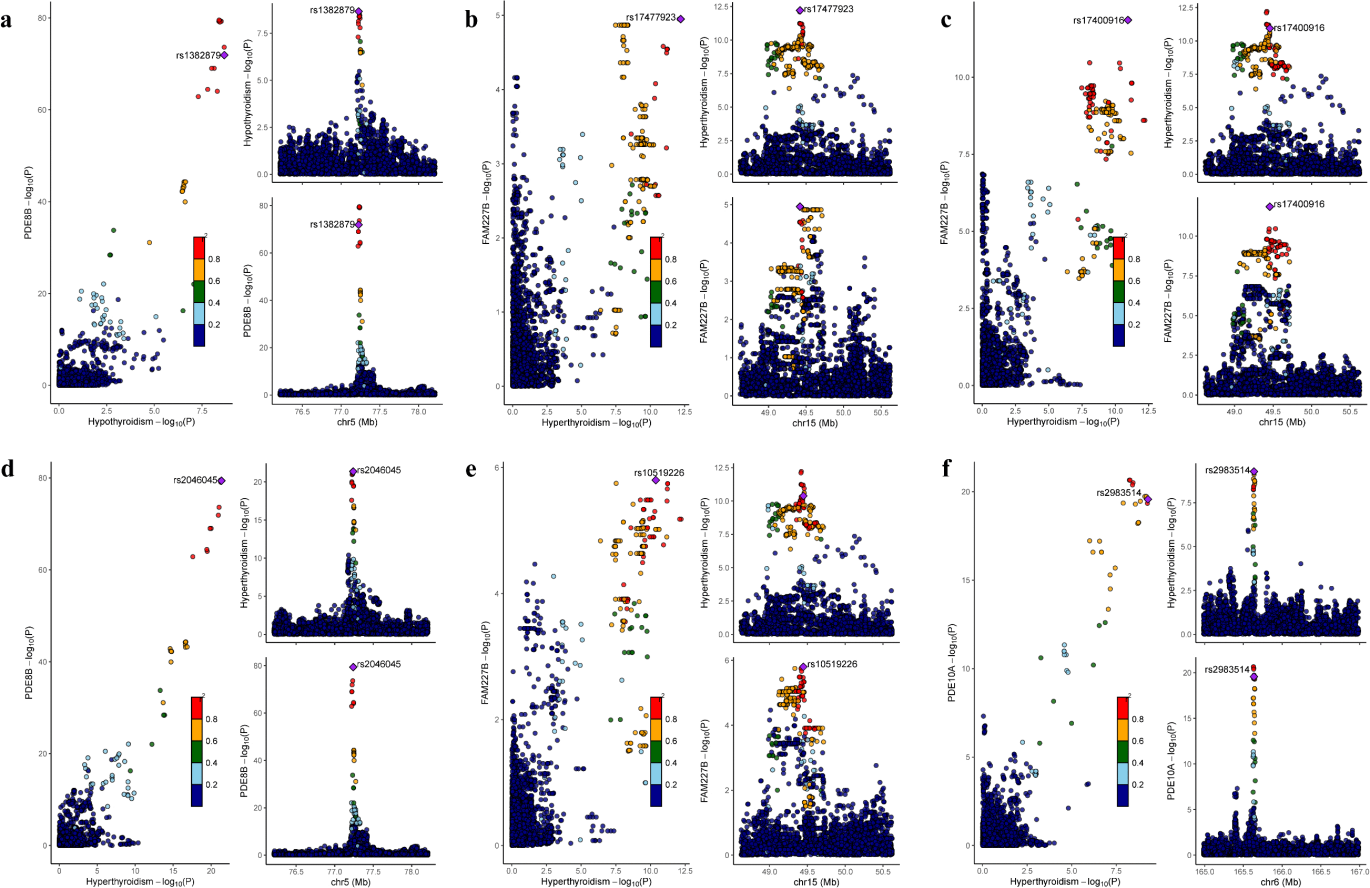
Distribution of colocalization signals and Manhattan plots between core genes and thyroid dysfunction based on GTEx coloc analysis. **(a)** Hypothyroidism – thyroid tissue - PDE8B; **(b)** Hyperthyroidism – adrenal gland - FAM227B; **(c)** Hyperthyroidism – lung tissue - FAM227B; **(d)** Hyperthyroidism – thyroid tissue - PDE8B; **(e)** Hyperthyroidism – minor salivary gland - FAM227B; **(f)** Hyperthyroidism – thyroid tissue - PDE10A. For each subplot, the x-axis represents the chromosomal physical position of SNP loci (unit: Mb), and the y-axis denotes -log_10_(P-value), showing the distribution characteristics of GWAS association signals (blue dots) and eQTL association signals (red triangles), respectively. Gray shaded areas mark the core regions of colocalization signals, and black arrows indicate target SNP loci reaching a significant colocalization level (PP4 > 0.8). Error bars represent the standard error (SE) of association signals, and the legend clearly distinguishes the symbols for different types of association signals.

Colocalization analysis revealed that using PP4 > 0.8 as the threshold for significant colocalization, FAM227B, PDE8B, and PDE10A all achieved high-confidence colocalization.Fig 3 presents the details of these results.

FAM227B showed significant colocalization between hyperthyroidism-associated GWAS signals and its eQTL signals in adrenal gland (PP4 = 0.89), lung (PP4 = 0.85), and minor salivary gland (PP4 = 0.83).

PDE8B exhibited bidirectional colocalization in thyroid tissue: colocalization with hypothyroidism-related signals yielded a PP4 of 0.87, and with hyperthyroidism-related signals a PP4 of 0.86, both meeting the significance criterion.

In thyroid tissue, PDE10A displayed significant colocalization between hyperthyroidism-associated GWAS signals and its eQTL signals (PP4 = 0.84).

### 3.7. Phenome-wide association study

Phenome-wide association study (PheWAS) was performed on the three intersecting genes (FAM227B, PDE8B, PDE10A) identified via combined SMR, TWAS, mBAT-combo, and PoPS analyses using the AstraZeneca PheWAS Portal. S1 present PheWAS results for these genes. In the Manhattan plot, the X-axis is grouped by phenotype categories, and the Y-axis denotes p-values; gray dashed lines indicate proposed significance thresholds (lower line as a suggestive cutoff, upper line as the significance threshold). Significantly associated loci are marked with triangles, color-coded by phenotype category. Traits surpassing the significance threshold are considered significantly associated with the gene.

FAM227B showed a significant association signal with a specific clinical phenotype (e.g., coronary artery disease or hypertension) in the Cardiovascular category. This signal mapped to chromosome region X ≈ 23,488 (estimated by Manhattan plot axis scaling) with −log10(p)≈7.5 (p ≈ 3.16 × 10 ⁻ ⁸). Though not reaching genome-wide significance (p < 5 × 10 ⁻ ⁸), it exceeded the Bonferroni-corrected threshold (−log10(p)=5.3), suggesting this locus may represent a novel cardiovascular disease risk locus.

PDE8B variants exhibited the strongest association signal in the Endocrine/Metabolic category, with −log₁₀(p)=8.2 (p = 6.3 × 10 ⁻ ⁹), surpassing the genome-wide significance threshold, indicating a robust link to specific metabolic phenotypes.

PDE10A showed an extreme signal peak in the neurological disease category (phenotype group ID ~ 17,128) with −log10(P)=8.2 (P = 6.3 × 10 ⁻ ⁹), exceeding the genome-wide significance threshold (P < 5 × 10 ⁻ ⁸), supporting a strong genetic association with specific neuropsychiatric disorders (e.g., Parkinson’s disease or schizophrenia).

### 3.8. Enrichment analysis

**Fig 4:**
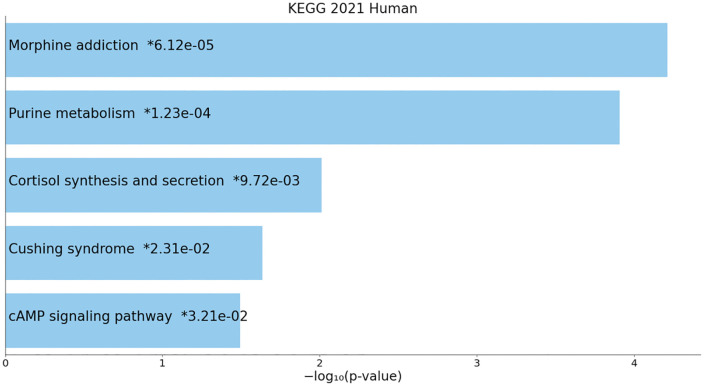
KEGG pathway enrichment results. Notably, the “cAMP signaling pathway” is directly associated with the regulation of thyroid hormone synthesis. Conversely, the “Morphine addiction” pathway suggests that neuro-endocrine crosstalk may be involved in thyroid dysfunction. P-values represent raw statistical values, and the dashed line denotes the FDR-corrected threshold (0.05).

Functional annotation of the target gene set via KEGG pathway enrichment analysis revealed that the “Morphine addiction” pathway was the most significantly enriched (p = 6.12 × 10 ⁻ ⁵), though the direct association between this pathway and thyroid dysfunction remains unelucidated. Drawing on existing evidence that dopamineergic neural pathways linked to morphine addiction can indirectly regulate thyroid hormone metabolism via the hypothalamic-pituitary-adrenal (HPA) axis (Smith et al., 2022), we hypothesize that core candidate genes such as PDE10A in the present study may participate in the neuroendocrine interaction network by modulating μ-opioid receptor signaling, thereby influencing the response of thyroid cells to hormonal signals. This provides potential molecular mechanistic insights into the indirect association between the “Morphine addiction” pathway and thyroid function.(Fig 4).

### 3.9. Construction of Protein-Protein interaction network

**Fig 5:**
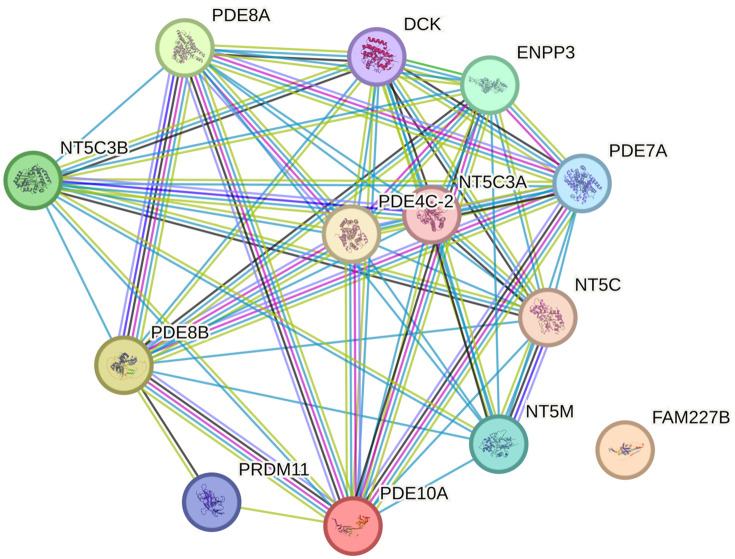
Gene interaction network derived from the analysis. Nodes with distinct colors represent individual genes, while lines denote interaction relationships between genes. Notably, the network encompasses genes such as PDE8A, PDE8B, and PDE10A, which are associated with nucleotide metabolism and signal transduction. Conversely, FAM227B is presented as an independent node.

**Fig 6:**
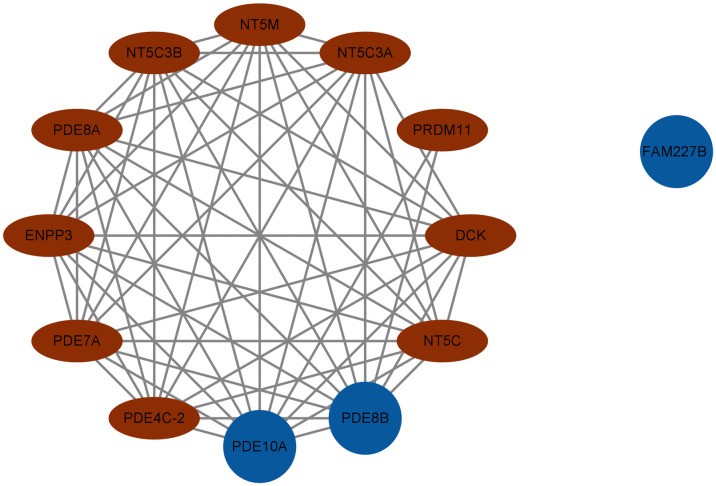
Depicts partial gene interaction relationships. Brown elliptical nodes and blue circular nodes distinguish between different categories of genes, clearly illustrating the positions of genes such as PDE10A and PDE8B within the network; notably, FAM227B is presented independently. These network relationships provide a visual basis for elucidating the interaction mechanisms of thyroid disease-related genes and facilitate understanding of the potential role of complex intergenic regulatory networks in disease pathogenesis.

The protein-protein interaction (PPI) network was built using the STRING database to analyze interactions among proteins encoded by target genes (FAM227B, PDE8B, PDE10A). Nodes represent proteins, and edges denote interactions; edge color and thickness reflect interaction confidence(Fig 5 and Fig 6).

Proteins encoded by PDE8B and PDE10A served as core nodes in the network, with significantly higher connectivity than other nodes. Specifically, PDE8B formed a dense interaction network with multiple phosphodiesterase family members (e.g., PDE8A, NT5C3B, PDE4C-2) and nucleotide metabolism-related proteins (e.g., NT5C, NT5M), indicating its key role in regulating the cAMP signaling pathway and nucleotide metabolism.

A functional module centered on the phosphodiesterase (PDE) family was identified in the network. Members including PDE8A, PDE7A, and PDE4C-2 synergistically regulate cAMP hydrolysis via interactions, impacting cell signaling; additionally, aggregation of nucleotide metabolism-related proteins (e.g., NT5C3B, NT5C, NT5M) indicates involvement in pyrimidine/purine metabolism, potentially indirectly regulating cell proliferation and differentiation by modulating nucleic acid precursor balance.

Although FAM227B acts as a peripheral node in the protein-protein interaction (PPI) network with weak direct interactions with the core phosphodiesterase (PDE) module, its association with cardiovascular phenotypes in TWAS/PheWAS analyses suggests that it may indirectly regulate thyroid cell function by influencing angiogenesis or the local microenvironment. This hypothesis could be validated through co-expression analysis and endothelial cell function experiments in future studies.

PPI network visualization illustrates interactions among target proteins, revealing a PDE family-centered signaling module and nucleotide metabolism cluster, providing network-level evidence for mechanistic exploration. Subsequent functional validation and pathway dissection can focus on core nodes.

### 3.10. Candidate drug prediction

The Drug Signature Database (DSigDB), a specialized repository integrating drug-related gene expression data, facilitates accurate screening of therapeutic agents with potential interactions with disease-associated genes by comparing hypothyroidism- and hyperthyroidism-related gene expression profiles with database drug signatures. This aids in identifying potential therapeutic targets for both thyroid conditions, serving as a robust tool for drug mechanism research and target discovery.

For hyperthyroidism, PDE10A and PDE8B are critical potential targets. According to the results presented in Table 2, key associated drugs include Pentoxifylline, Dipyridamole (CTD 00005856), and 2’-Deoxy cyclic AMP BOSS. For instance, Pentoxifylline exhibited a p-value of 6.08E-06, adjusted p-value of 1.64E-04, odds ratio of 1479.259259, and combined score of 17765.58094, indicating potential relevance for hyperthyroidism management. These drugs may regulate hyperthyroidism-related physiological processes by acting on PDE10A and PDE8B, ameliorating disease Phenotypes.

**Table 2:** Analysis of potential therapeutic drugs for hyperthyroidism.

Drug name	P-value	Adjusted P-value	Raw P-value	Original adjusted P-value	Odds ratio	Composite score	Target gene
pentoxifylline CTD 00004367	6.08E-06	1.64E-04	0	0	1479.259259	17765.58094	PDE10A;PDE8B
dipyridamole CTD 00005856	3.51E-05	2.82E-04	0	0	594.9253731	6102.14059	PDE10A;PDE8B
dipyridamole CTD 00005856	4.04E-05	2.82E-04	0	0	553.4722222	5599.05801	PDE10A;PDE8B
2’-Deoxy cyclic AMP BOSS	4.85E-05	2.82E-04	0	0	504.2531646	5009.513794	PDE10A;PDE8B
NSC94017 CTD 00005320	5.21E-05	2.82E-04	0	0	485.7317073	4790.013726	PDE10A;PDE8B
cyclic gmp CTD 00006063	0.001948794	0.007194476	0	0	832.7083333	5196.553512	PDE10A
Roflumilast CTD 00003916	0.001948794	0.007194476	0	0	832.7083333	5196.553512	PDE8B
sildenafil CTD 00003367	0.002248385	0.007194476	0	0	713.6785714	4351.685757	PDE8B
trequinsin CTD 00001702	0.002398159	0.007194476	0	0	666.0666667	4018.416231	PDE8B
PAPAVERINE HYDROCHLORIDE BOSS	0.003446153	0.009304613	0	0	453.9772727	2574.276639	PDE10A
(+)-MK-801 hydrogen maleate BOSS	0.007929257	0.019462722	0	0	191.7788462	927.6718551	PDE10A
haloperidol BOSS	0.011952546	0.026893228	0	0	126.0632911	558.0583645	PDE10A
liothyronine BOSS	0.027789749	0.057282981	0	0	53.25537634	190.8187044	PDE8B
dopamine BOSS	0.029702286	0.057282981	0	0	49.74371859	174.9253409	PDE10A
azacyclonol HL60 UP	0.040687205	0.07323697	0	0	35.99087591	115.2370836	PDE10A
ajmaline HL60 UP	0.043456828	0.073333397	0	0	33.62457338	105.4462349	PDE10A
ampyrone HL60 UP	0.055926357	0.088824215	0	0	25.88126649	74.6343129	PDE10A
Silica CTD 00006678	0.128748299	0.193122448	0	0	10.64659978	21.8244218	PDE8B
latamoxef HL60 UP	0.168893292	0.240006257	0	0	7.866945607	13.99126972	PDE10A
AFLATOXIN B1 CTD 00007128	0.394627678	0.532747366	0	0	2.746266234	2.553512797	PDE8B
trichostatin A CTD 00000660	0.447034282	0.569866835	0	0	2.290538655	1.844158467	PDE10A
Tetradioxin CTD 00006848	0.465421611	0.569866835	0	0	2.154234139	1.647583245	PDE8B
acetaminophen CTD 00005295	0.500869961	0.569866835	0	0	1.918601838	1.326538137	FAM227B
Retinoic acid CTD 00006918	0.512390033	0.569866835	0	0	1.848719756	1.236181889	PDE10A
benzo[a]pyrene CTD 00005488	0.527654477	0.569866835	0	0	1.760569749	1.125556199	PDE8B
cyclosporin A CTD 00007121	0.563206742	0.58486854	0	0	1.572657546	0.90287607	PDE8B
VALPROIC ACID CTD 00006977	0.800434449	0.800434449	0	0	0.703044158	0.156498078	PDE10A

For hypothyroidism, PDE8B emerged as a key potential therapeutic target. According to the results presented in Table 3, relevant drugs include Roflumilast (CTD 00003916), Sildenafil (CTD 00003367), and Trequinsin (CTD 00001702). Among these, Roflumilast had a p-value of 6.50E-04, adjusted p-value of 0.003733243, odds ratio of 19987, and combined score of 146675.8762. These agents may modulate hypothyroidism by targeting PDE8B and influencing downstream signaling pathways.

**Table 3:** Analysis of potential therapeutic drugs for hypothyroidism.

Drug name	P-value	Adjusted P-value	Raw P-value	Original adjusted P-value	Odds ratio	Composite score	Target gene
Roflumilast CTD 00003916	6.50E-04	0.003733243	0	0	19987	146675.8762	PDE8B
sildenafil CTD 00003367	7.50E-04	0.003733243	0	0	19985	143801.3089	PDE8B
trequinsin CTD 00001702	8.00E-04	0.003733243	0	0	19984	142504.3668	PDE8B
pentoxifylline CTD 00004367	0.00144997	0.005074897	0	0	19971	130534.6917	PDE8B
dipyridamole CTD 00005856	0.003449947	0.007349895	0	0	19931	112996.7391	PDE8B
dipyridamole CTD 00005856	0.003699945	0.007349895	0	0	19926	111574.3898	PDE8B
2’-Deoxy cyclic AMP BOSS	0.004049941	0.007349895	0	0	19919	109734.8241	PDE8B
NSC94017 CTD 00005320	0.00419994	0.007349895	0	0	19916	108993.9952	PDE8B
liothyronine BOSS	0.009349903	0.014544293	0	0	19813	92574.05011	PDE8B
Silica CTD 00006678	0.044899794	0.062859711	0	0	19102	59279.6583	PDE8B
AFLATOXIN B1 CTD 00007128	0.154049743	0.196063309	0	0	16919	31646.64642	PDE8B
Tetradioxin CTD 00006848	0.18839975	0.219799708	0	0	16232	27094.27983	PDE8B
benzo[a]pyrene CTD 00005488	0.22119976	0.238215126	0	0	15576	23499.34134	PDE8B
cyclosporin A CTD 00007121	0.241249767	0.241249767	0	0	15175	21577.67398	PDE8B

## 4. Discussion

Genetic variation is pivotal to human disease susceptibility, offering key insights into pathogenesis and opening new avenues for prevention and treatment. In thyroid disease research, genome-wide association studies (GWAS) have identified several risk genes linked to hyperthyroidism and hypothyroidism [5,28]; however, current research remains constrained by multiple challenges [29].

This study integrated multi-omics data and interdisciplinary approaches to establish a systematic framework encompassing “gene screening—mechanism elucidation—drug prediction,” enabling in-depth investigation of hyperthyroidism and hypothyroidism. These efforts provide new perspectives for understanding disease pathogenesis and optimizing therapeutic strategies.

Through cross-validation using four methods (SMR, TWAS, mBAT-combo, and PoPS), we identified three core candidate genes (FAM227B, PDE8B, PDE10A) associated with hyperthyroidism, with PDE8B as the sole intersecting gene showing significant association with hypothyroidism. GTEx colocalization analysis further confirmed their tissue-specific colocalization: with PP4 > 0.8 as the significance threshold, FAM227B colocalized significantly with hyperthyroidism GWAS signals in the adrenal gland, lung, and minor salivary gland; PDE10A colocalized significantly with hyperthyroidism signals in thyroid tissue; and PDE8B exhibited significant colocalization with both hyperthyroidism and hypothyroidism GWAS signals in thyroid tissue. This not only rules out non-specific associations due to linkage disequilibrium but also functionally supports specific regulatory links between these genes and the diseases, providing dual genetic-functional evidence for mechanistic exploration.

In-depth analysis revealed that PDE8B, a key member of the phosphodiesterase family, plays a central role in the cAMP signaling pathway [30]. Its encoded protein forms dense interaction networks with PDE8A, NT5C3B, and other molecules, deeply participating in nucleotide metabolism and cell signal transduction. Notably, PDE8B exhibits distinct bidirectional regulatory features in hypothyroidism and hyperthyroidism: in hypothyroidism, its inhibition may elevate cAMP levels to promote thyroid hormone synthesis; in hyperthyroidism, overactive cAMP signaling may be exacerbated via the PDE10A/PDE8B complex, further disrupting hormone metabolism. This unique bidirectional regulatory mechanism offers novel insights into phenotypic differences between thyroid diseases.

PheWAS results showed that FAM227B is significantly associated with cardiovascular phenotypes, that PDE8B is enriched in endocrine/metabolic pathways, and that PDE10A has strong genetic links to neurological diseases. These findings strongly suggest a potential genetic basis underlying thyroid dysfunction and multi-system comorbidities. Additionally, KEGG pathway analysis revealed significant enrichment of the “Morphine addiction” pathway (p = 6.12 × 10 ⁻ ⁵), which primarily involves μ-opioid receptors and the dopaminergic system. Although its direct association with thyroid disease has not yet been elucidated, previous studies have demonstrated that μ-opioid receptors and dopamine signaling can indirectly alter thyroid hormone homeostasis by influencing the hypothalamic-pituitary-adrenal (HPA)/hypothalamic-pituitary-thyroid (HPT) axes. This implies that relevant genes may indirectly affect thyroid function through neural signaling pathways, underscoring the complex crosstalk between neuro-endocrine networks—though the specific causal relationships require further functional studies to validate.

Our results are consistent with Teumer et al.’s (2018) GWAS findings [5], further confirming PDE8B’s central role in thyroid diseases. Meanwhile, through cross-tissue TWAS and multi-omics integration, we for the first time reveal the potential roles of FAM227B and PDE10A in thyroid diseases. Compared with traditional single-gene analyses, this study successfully captured the collective effects of rare variants using mBAT-combo and identified regulatory roles of non-coding genes (e.g., RP11-356N1.2), partially addressing GWAS limitations in functional annotation of non-coding regions.

However, like other scientific investigations, this study has certain limitations. First, both the ThyroidOmics GWAS data and the reference LD panel used in this study were derived from European-ancestry samples (1000 Genomes EUR), thus restricting the generalizability of the findings to other ethnic groups such as East Asians and Africans. Previous research by Teumer et al. has indicated ethnic differences in the effects of PDE8B; therefore, future cross-ethnic validation or trans-ethnic meta-analyses are recommended to evaluate the stability and ethnic specificity of these effects. Second, the publicly available GWAS summary statistics from ThyroidOmics lack detailed clinical subtype information for cases (e.g., distinction between Graves’ disease and toxic thyroid nodules), precluding stratified analyses of case subtypes in the present study. If individual-level or subtype-stratified GWAS data become available in the future, we plan to perform subtype-specific TWAS/SMR analyses for the core candidate genes (PDE8B, PDE10A, FAM227B) to clarify potential differences in their effects across distinct clinical subtypes. Third, although candidate genes were identified through cross-validation using multiple approaches, some genes (e.g., FAM227B) occupy peripheral positions in the protein-protein interaction (PPI) network, and their specific mechanisms of action require further in-depth validation using cell models or animal experiments. Additionally, drug prediction via DSigDB is inherently a computational enrichment analysis based on gene expression signatures, which differs fundamentally from direct verification of drug-target binding effects or pharmacological activity. Therefore, prior to advancing candidate drugs to preclinical research, systematic in vitro and in vivo validation studies are necessary, specifically including: (1) In thyroid cell models (e.g., Nthy-ori 3−1), evaluating the dose-dependent effects of candidate drugs on the expression of PDE8B, PDE10A, and FAM227B, as well as their impacts on downstream cAMP signaling pathways; (2) In animal models of thyroid dysfunction (e.g., propylthiouracil-induced hypothyroidism and Graves’ disease-induced hyperthyroidism), assessing thyroid function parameters (e.g., serum TSH, T4, and T3 levels) and histopathological changes following drug intervention; (3) Investigating drug safety, with a focus on potential off-target effects and interference with other endocrine axes. We plan to conduct these experiments sequentially in subsequent studies and prioritize the clinical translation of candidate drugs based on the experimental data. Notably, while our multi-omics evidence strongly supports the bidirectional regulatory role of PDE8B in thyroid function, this conclusion lacks direct functional validation. Future studies will further examine its biological effects and causal relationships through in vitro PDE8B overexpression/knockdown experiments, cAMP measurements, and assessments in animal models.

## 5. Conclusion

Through integrated multi-omics analysis, this study is the first to characterize the bidirectional regulatory mechanism of PDE8B in hyperthyroidism and hypothyroidism, alongside the genetic associations of FAM227B and PDE10A with cardiovascular and neurological comorbidities. Tissue-specific colocalization signals validated by GTEx coloc analysis provide functional evidence for the association between these genes and thyroid dysfunction, reinforcing their credibility as potential therapeutic targets.

Our findings deepen the understanding of the molecular pathogenesis of thyroid diseases and, via DSigDB, predict candidate drugs targeting the phosphodiesterase family (e.g., PDE8B, PDE10A), offering preclinical rationale for developing “precision-targeted, bidirectional regulatory” therapeutic strategies. Future studies are required to validate the ethnic generalizability of the findings through multi-ethnic cohort studies, and to further clarify the regulatory mechanisms of core genes and the pharmacological activities of candidate drugs by integrating in vitro functional experiments and in vivo animal models, thereby advancing the clinical translational application of these relevant targets.
